# Numerical treatment of radiative Nickel–Zinc ferrite-Ethylene glycol nanofluid flow past a curved surface with thermal stratification and slip conditions

**DOI:** 10.1038/s41598-020-73720-x

**Published:** 2020-10-08

**Authors:** Muhammad Ramzan, Nosheen Gul, Jae Dong Chung, Seifedine Kadry, Yu-Ming Chu

**Affiliations:** 1 Department of Computer Science, Bahria University, Islamabad Campus, Islamabad, 44000 Pakistan; 2 Department of Mechanical Engineering, Sejong University, Seoul, 143-747 Korea; 3 Department of Mathematics and Computer Science, Faculty of Science, Beirut Arab University, Beirut, 115020 Lebanon; 4 Department of Mathematics, Huzhou University, Huzhou, 313000 People’s Republic of China; 5 Hunan Provincial Key Laboratory of Mathematical Modeling and Analysis in Engineering, Changsha University of Science and Technology, Changsha, 410114 People’s Republic of China

**Keywords:** Applied mathematics, Mechanical engineering

## Abstract

The inadequate cooling capacity of the customary fluids forced the scientists to look for some alternatives that could fulfill the industry requirements. The inception of nanofluids has revolutionized the modern industry-oriented finished products. Nanofluids are the amalgamation of metallic nanoparticles and the usual fluids that possess a high heat transfer rate. Thus, meeting the cooling requirements of the engineering and industrial processes. Having such amazing traits of nanofluids in mind our aim here is to discuss the flow of nanofluid comprising Nickel–Zinc Ferrite and Ethylene glycol over a curved surface with heat transfer analysis. The heat equation contains nonlinear thermal radiation and heat generation/absorption effects. The envisioned mathematical model is supported by the slip and the thermal stratification boundary conditions. Apposite transformations are betrothed to obtain the system of ordinary differential equations from the governing system in curvilinear coordinates. A numerical solution is found by applying MATLAB build-in function bvp4c. The authentication of the proposed model is substantiated by comparing the results with published articles in limiting case. An excellent concurrence is seen in this case. The impacts of numerous physical parameters on Skin friction and Nusselt number and, on velocity and temperature are shown graphically. It is observed that heat generation/absorption has a significant impact on the heat transfer rate. It is also comprehended that velocity and temperature distributions have varied behaviors near and far away from the curve when the curvature is enhanced.

## Introduction

The customary fluids including oil, and ethylene, etc. possess a low heat transfer rate. This rate is doubled once metallic nanoparticles sized (< 100 nm) are inserted with a ratio (< 1%) into the base fluids. The thermal conductivity of the ordinary fluids is affected by the numerous impacts comprising volume fraction, temperature, nature of the particles, and the dimensions of the metallic particles. These material particles may be in the form of metals, oxides, and carbides with distinctive chemical and physical characteristics. Choi and Eastman^1^ incepted the novel idea of nanofluids. The applications associated with nanofluids include numerous fields like manufacturing, transportation, medical, defense, and acoustics, etc. Two renowned nanofluid models namely “Buongiorno” and “Tiwari and Das” are adopted in the literature by scientists. The former highlights the Brownian and thermophoretic impacts of the nanofluid flow. Nevertheless, the later highlights the characteristics of the metallic particles inserted into the base fluid. Here, we have adopted the “Tiwari and Das” nanofluid model. Lately, Nadeem et al.^2^ numerically explored the hybrid nanofluid flow comprising Copper and Aluminum oxide nanoparticles and the water over an exponentially stretched curved surface. The study revealed that the rate of heat transfer is higher in the case of hybrid nanofluid in comparison to the simple base fluid. The unsteady Sisko nanofluid flow with thermal radiation and the Hall effect is examined by Ali et al.^3^. The key outcome of this study is that higher curvature of the curve boosts the fluid velocity. Acharya et al.^4^ discussed the flow of the nanofluid with carbon nanotubes inserted into water influenced by the mixed convection and the slip condition at the boundary of the curved surface. It is comprehended from this exploration that fluid temperature is decreased once the curvature of the curved surface is enhanced. The impact of hybrid nanofluid flow over a shrinking/stretching surface with stability analysis is examined numerically by Waini et al.^5^. Mir et al.^6^ numerically handled the flow of hybrid nanofluid with silver/water combination over an elliptically curved channel. The results obtained highlight that enhancement in nanoparticle volume fraction boosts the fluid temperature. The three-dimensional flow of varied combinations of nanofluid inside a vertical channel wall is examined by Gholami et al.^7^. The outcome of this exploration states that the fluid friction factor is enhanced with an increase in nanoparticle volume fraction. He et al.^8^ studied the nanofluid flow with a twisted tape inserted in a tube. It is concluded here that the use of one twisted tape possesses more thermal fluid performance when compared with two twisted tapes. The flow of nanofluid with numerous nanoparticles inserted into the base fluid amid two parallel disks under the influence of suction/injection and viscous and ohmic dissipations is analyzed by Dogonchi et al.^9^. The significant upshot of the present investigation is that velocity and the temperature profiles show an opposing trend for the suction/injection parameter. More studies highlighting numerous aspects of nanofluids may be found at^10–21^.

The appropriate dispersion of nanoparticles causes remarkable enhancement in the thermal conductivity of the customary fluid. The ferrite nanoparticles mixed in the base fluid enhances the thermal conductivity and heat transfer capability of the customary fluid. and. There are many worth mentioning examples of heat transfer such as avionics cooling systems and cooling/heating of buildings. The large surface area of nanoparticles in comparison to micrometer-sized particles, qualify them with unmatched heat transfer qualities^22^. The utilization of Nickel–zinc ferrite can be seen in electromagnetic applications with higher permeability like inductors and electromagnetic wave absorbers. Many researchers have recommended that the use of Nickel-zinc nanoparticles can minimize the energy losses associated with bulk powders^23–25^. In ferromagnetic nanofluids hyperthermia, ferrites nanoparticles of various types including MnZnFe_2_O_4_, Fe_2_O_4_, and NiZn-Fe_2_O_4_ are infused in tumor and are subjected under a high-frequency magnetic field. These ferrite nanoparticles produce heat that regularly enhances tumor temperature, which can kill cancer cells^26^. Some recent explorations highlighting the impact of numerous nanoparticles include work by Ramzan et al.^27^ who explored numerically the nanofluid flow with the insertion of carbon nanotubes between two parallel disks. The flow is assisted by the impacts of modified Fourier law in a Darcy-Forchheimer permeable media. It is observed that the fluid velocity and the temperature show opposing effects for the local inertial coefficient. Karbasifar et al.^28^ deliberated the nanofluid flow containing Aluminum oxide and water in a lid-driven cavity inclined at an angle with a hot cylinder inside with mixed convection. The main outcome of the study revealed that higher estimates of the volume fraction and Reynolds number boost the Nusselt number. The heat transfer impact os the water and functional multi-walled carbon nanotubes based nanofluid flow in a backward-facing channel is numerically handled by Alrashed et al.^29^. The chief result of the exiting model is that the vortex will be closer to the opening of the channel for large Reynolds number. Recent studies on heat transfer of nanofluid flow with nanoparticles inserted into some base fluid may be found in^30–45^.

The curved stretching, in modernized engineering technologies, has enormous significance and applications *e.g.*, in the transportation sector and electronics. Studies featuring fluid flows over the curved surfaces are discussed in the literature in numerous ways. Sanni et al.^46^ found the numerical solution for a viscous liquid flow over a nonlinearly curved stretched channel. The numerical investigation of MHD nano liquid flow along with the heat transit over a nonlinearly stretched curved sheet is conducted by Sharma et al.^47^. Afridi et al.^48^ using the second law of thermodynamics analyzed heat transfer with influences of the magnetic field, dissipation, and entropy production in nano liquid flow past a curved stretching sheet. Ferro-fluid flow through an extended curved surface is demonstrated by Sajid et al.^49^ considering the magnetic forces and Joule heating. The time-dependent fluid flow through a curved spongy sheet is analyzed by Rosca and Pop^50^. The flow of nanofluid past a linearly curved stretched surface with entropy optimization and nonlinear radiation is examined by Lu et al.^51^. The radiation effects of MHD nano liquid flow over a curved sheet is examined by Abbas et al.^52^, in the existence of heat generation and Lorentz force by considering the slip effect.

Given the foregoing, it is revealed from the above-cited literature that there are a good number of studies considering linear/nonlinear/exponential stretched surfaces. But fewer studies are reported that discusses the nanofluid flows over curved surfaces. Here, in this study, the novelty lies in the numerical solution of the flow of nanofluid comprising Nickel–Zinc Ferrite and Ethylene glycol over a curved surface with heat transfer analysis. The heat equation comprises nonlinear thermal radiation and heat generation/absorption effects. The proposed mathematical model is reinforced by the slip and the thermal stratification boundary conditions. To our knowledge, no such study is carried out that highlights all such aspects. All outcomes of the present exploration are depicted through the graphical illustrations, and in numerically erected tables.

## Description of mathematical formulation

**Figure 1:**
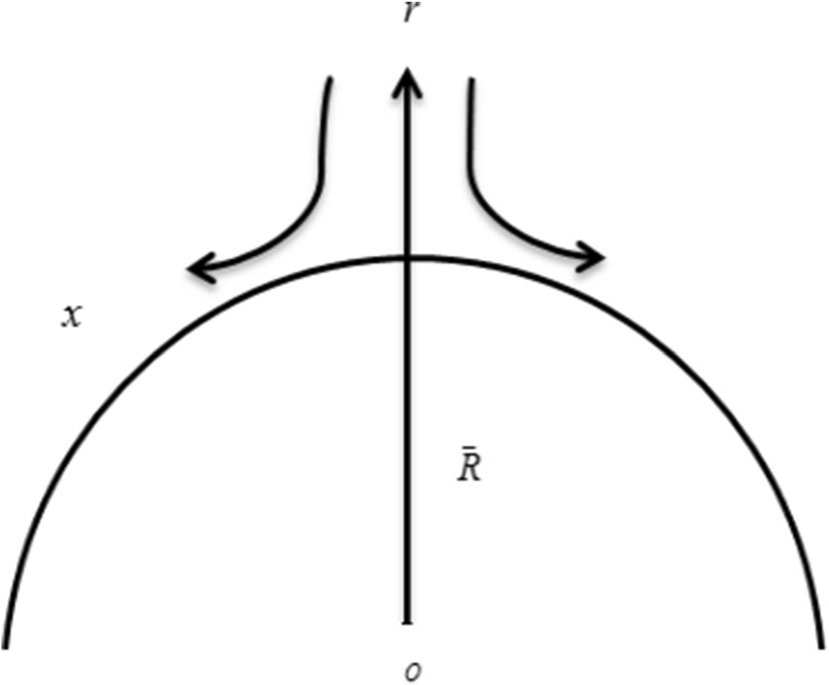
Flow geometry of a mathematical model for a curved surface.

Consider a time-independent 2D, isochoric boundary layer flow of nano liquid past a curved stretchable surface. In the presence of nonlinear heat-flux and heat source, flow analysis is adopted with thermal stratification. A curvilinear coordinate system is adopted in such a manner that *x*-axis is directed along a curved stretching surface whereas the *r*-axis is normal to the *x-*axis. Here $U_{W} = Sx$ , where $S$ is the positive real number, is the linearly stretchable velocity with distance from the origin, and $\bar{R}$ is the radius of the curved surface. In the *r*-direction, an invariable magnetic field is applied (Fig. 1). Assuming the Reynolds number to be very small, the effect of the induced and electric–magnetic field can be ignored.

The subsequent continuity, monument and energy equations^51, 52^ govern the assumed system:

1 $$((\bar{R} + r)V)_{r} + \bar{R}U_{x} = 0,$$

2 $$\frac{{U^{2} }}{{r + \bar{R} }} = \frac{1}{{\rho_{nf} }}p_{r} ,$$

3 $$VU_{r} + \frac{{\bar{R}}}{{r + \bar{R}}}UU_{x} + \frac{1}{{r + \bar{R}}}UV = - \frac{{\bar{R}}}{{(r + \bar{R})\rho_{nf} }}p_{x} + \frac{{\mu_{nf} }}{{\rho_{nf} }}\left(U_{rr} + \frac{1}{{r + \bar{R}}}U_{r} - \frac{1}{{(r + \bar{R})^{2} }}U\right) - \frac{{\sigma^{*} \beta_{0}^{2} }}{{\rho_{nf} }}U,$$

4 $$\begin{aligned} VT_{r} + \frac{{\bar{R}}}{{r + \bar{R}}}UT_{x} & = \alpha (T_{rr} + \frac{1}{{r + \bar{R}}}T_{r} ) + \frac{1}{{\left( {\rho C_{p} } \right)_{nf} }}(T - T_{\infty } )Q^{*} \\ & \quad + \frac{1}{{(r + \bar{R})\left( {\rho C_{p} } \right)_{nf} }}((r + \bar{R})q_{r} )_{r} . \\ \end{aligned}$$

The associated boundary conditions are:

5 $$\begin{aligned} & \left. V \right|_{r = 0} = 0,\, \, \left. U \right|_{r = 0} = U_{W} (x) + L\left( {U_{r} + \frac{1}{{r + \bar{R} }}U} \right),\,\left. T \right|_{r = 0} = T_{W } = T_{0} + n_{1} x \\ & \left. U \right|_{r \to \infty } \to 0,\, \, \left. {U_{r} } \right|_{r \to \infty } \to 0, \, \left. T \right|_{r \to \infty } = T_{\infty } = T_{0} + n_{2} x. \\ \end{aligned}$$

The thermophysical traits of the nano-liquid are mentioned in Table 1.

**Table 1:** The estimated values of the thermophysical characteristics (of ρ, $k$, $C_{p}$)^53^ of the Ethylene glycol and Nickel-zinc ferrite

Thermophysical properties	Ethylene glycol (C_2_H_6_O_2_)	Nickel–Zinc ferrite (NiZnFe_2_O_4_)
C_p_ (J kg^−1^ k^−1^)	2382	710
$\rho$ (kg m^−3^)	1116.6	4800
$k$ (W m^−1^ K^-1^)	0.249	6.3
Pr	204	–

These properties are represented in the mathematical form as^53^:

$$\mu_{nf} = \mu_{f} (1 - \psi )^{ - 2.5} ,\,\;\alpha = \frac{{k_{nf} }}{{(\rho C_{p} )_{nf} }},$$

$$\frac{{\rho_{nf} }}{{\rho_{f} }} = (1 - \psi ) + \psi \frac{{\rho_{p} }}{{\rho_{f} }}, \, \frac{{(\rho C_{p} )_{nf} }}{{(\rho C_{p} )_{f} }} = (1 - \psi ) + \psi \frac{{(\rho C_{p} )_{p} }}{{(\rho C_{p} )_{f} }},$$

6 $$\frac{{k_{nf} }}{{k_{f} }} = \frac{{(k_{p} + 2k_{f} ) + 2\psi (k_{p} \, - k_{f} )}}{{(k_{p} + 2k_{f} ) - \psi (k_{p} \, - k_{f} )}}.$$

Nonlinear radiation heat-flux (under Rosseland approximation) used in Eq. (4), is as below:

7 $$q_{r} = \frac{{4\bar{\sigma }}}{{3\bar{k}}}(T^{4} )_{r} = \frac{{16\bar{\sigma }}}{{3\bar{k}}}T^{3} T_{r}$$

## Solution procedure

Here, the subsequent dimensionless transformations are used:

8 $$\begin{aligned} & \xi = \left( {\frac{S}{{\upsilon_{f} }}} \right)^{\frac{1}{2}} r,\,p = \rho_{f} S^{2} x^{2} P(\xi ),\,{\text{U}} = SxF^{{\prime }} (\xi ),\,\Theta = \frac{{T - T_{\infty } }}{{T_{W} - T_{\infty } }}, \\ & {\text{V}} = - \frac{{\bar{R} }}{{r + \bar{R} }}\sqrt {S\upsilon_{f} } F\left( \xi \right), \, T = T_{\infty } \left( {1 + (\Theta_{W} - 1)\Theta } \right). \\ \end{aligned}$$

Here, the superscript (${{\prime }}$) indicates the derivative with respect ξ and $\Theta_{W} = \frac{{T_{W} }}{{T_{\infty } }}$. From the above transformations (8), the satisfaction of Eq. (1) is inevitable. However, Eqs. (2)–(5) reduce to:

9 $$P^{{\prime }} = \left( {1 - \psi + \psi \frac{{\rho_{s} }}{{\rho_{f} }}} \right)\frac{{F^{\prime 2} }}{{\xi + k_{c} }},$$

10 $$\begin{aligned} \frac{{2k_{c} }}{{\xi + k_{c} }}P & = \left( {1 - \psi + \psi \frac{{\rho_{s} }}{{\rho_{f} }}} \right)\left( {\frac{{k_{c} }}{{k_{c} + \xi }}(FF^{{\prime \prime }} - F^{\prime 2} ) + \frac{{k_{c} }}{{(k_{c} + \xi )^{2} }}FF^{{\prime }} - MF^{{\prime }} } \right) \\ & \quad + (1 - \psi )^{ - 2.5} \left( {F^{{{\prime \prime \prime }}} + \frac{{F^{{\prime \prime }} }}{{\xi + k_{c} }} - \frac{{F^{{\prime }} }}{{(\xi + k_{c} )^{2} }}} \right), \\ \end{aligned}$$

11 $$\begin{aligned} & \frac{1}{\Pr }\left( {\frac{{k_{nf} }}{{k_{f} }} + Ra(1 + (\Theta_{W} - 1)\Theta )^{3} } \right)\left( {\Theta^{{\prime \prime }} + \frac{1}{{\xi + k_{c} }}\Theta^{{\prime }} } \right) + 3Ra(1 + (\Theta_{W} - 1)\Theta )^{2} (\Theta_{W} - 1)\Theta^{{{\prime }2}} \\ & \quad + \lambda^{*} \Theta + (1 - \psi + \psi \frac{{(\rho C_{p} )_{p} }}{{(\rho C_{p} )_{f} }})\left( {\frac{{k_{c} }}{{\xi + k_{c} }}\left( {F\Theta^{{\prime }} + F^{{\prime }} \Theta + \frac{{S_{t} }}{{1 - S_{t} }}F^{{\prime }} } \right)} \right) \, = 0, \\ \end{aligned}$$

and

12 $$\begin{aligned} & {\text{F}}(\xi ) = 0,\quad {\text{F}}^{{\prime }} (\xi ) = 1 + \kappa \left( {F^{{\prime \prime }} + \frac{1}{{k_{c} }}F^{{\prime }} } \right),\quad \Theta^{{\prime }} (\xi ) = 1 - S_{t} ,\quad {\text{as}}\quad \xi = 0, \\ & F^{{\prime }} (\xi ) \to 0,\quad {\text{F}}^{{\prime \prime }} (\xi ) \to 0, \, \Theta (\xi ) \to 0,\,{\text{as}}\,\xi \to \infty . \\ \end{aligned}$$

Excluding the pressure term P(ξ) by solving Eqs. (9) and (10), resulting in the following equation:

13 $$\begin{aligned} & F^{iv} + \tfrac{2}{{\xi + k_{c} }}F^{{{\prime \prime \prime }}} - \tfrac{1}{{(\xi + k_{c} )^{2} }}F^{{\prime \prime }} + \tfrac{1}{{(\xi + k_{c} )^{3} }}F^{{\prime }} + \left( {1 - \psi } \right)^{2.5} \left( {1 - \psi + \psi \frac{{\rho_{s} }}{{\rho_{f} }}} \right)\left\{ \tfrac{{k_{c} }}{{\xi + k_{c} }}(F^{{\prime }} F^{{\prime \prime }} - FF^{{{\prime \prime \prime }}} ) \right.\\ & \quad \left. - \tfrac{{k_{c} }}{{(\xi + k_{c} )^{2} }}(F^{\prime 2} - FF^{{\prime \prime }} ) - \tfrac{{k_{c} }}{{(\xi + k_{c} )^{3} }}FF^{{\prime }} \right\} - \left( {1 - \psi } \right)^{2.5} M\left( {F^{{\prime \prime }} + \tfrac{1}{{\xi + k_{c} }}F^{{\prime }} } \right) = 0, \\ \end{aligned}$$

With

14 $$\begin{aligned} & k_{c} = \bar{R} \left( {\frac{S}{{\upsilon _{f} }}} \right),S_{t} = \frac{{n_{1} }}{{n_{2} }},\kappa = \frac{{Ls}}{{\upsilon _{f} }},Ra = \frac{{16\bar{\sigma } T_{\infty } ^{3} }}{{3k_{f} \bar{k} }},M = \sigma *\frac{{\beta _{0} }}{{\rho _{f} S}} \\ & \quad \lambda ^{*} = \frac{{Q^{*} }}{{S(\rho C_{p} )_{f} }}\,{\text{and}}\,\Pr = \frac{{\upsilon _{f} }}{\alpha }. \\ \end{aligned}$$

The mathematical expression of $C_{fx}$ and $Nu_{x}$ are appended as follows:

15 $$C_{fx} = \frac{{\tau_{rx} }}{{\tfrac{1}{2}\rho U_{W}^{2} }},\quad Nu_{x} = \frac{{xq_{w} }}{{k_{f} (T_{W} - T_{\infty } )}},$$

where heat flux and shear stress of the wall is given as:

16 $$\tau_{rx} = \mu_{nf} \left. {(U_{r} - \frac{1}{r + R}U)} \right|_{r = 0} , \, q_{W} = \left. {(q_{r} - k_{nf} T_{r} )} \right|_{r = 0} , \,$$

The transformations (8) with (16) formulate Eq. (15) as:

17 $$\begin{aligned} & \frac{1}{2}(Re_{x} )^{0.5} C_{f} = \left( {1 - \psi } \right)^{ - 2.5} \{ F^{{\prime \prime }} (0) - \frac{1}{{k_{c} }}F^{{\prime }} (0)\} , \, \\ & \quad (Re_{x} )^{ - 0.5} Nu_{x} = - \left[ {\frac{{k_{nf} }}{{k_{f} }} + R_{a} (1 + (\Theta_{W} - 1)\Theta (0))^{3} } \right]\Theta^{{\prime }} (0), \\ \end{aligned}$$

Here, Reynolds number is given as:

18 $${\text{Re}}_{x} = \frac{{U_{W} }}{{\upsilon_{f} }}x.$$

## Numerical solution

The nonlinear DEs (Differential equations) are numerically evaluated for better comprehension of the problem. The nonlinear ODEs (11) and (13) are solved numerically implementing MATLAB function $bvp4c$ and using given boundary conditions given in Eq. (12). We consider $F = y(1),{\text{ F}}^{{\prime }} = y(2),{\text{ F}}^{{\prime \prime }} = y(3),{\text{ F}}^{{{\prime \prime \prime }}} = y(4),{\text{ F}}^{iv} = yy1,$ $\Theta = y(5), \, \Theta ^{\prime} = y(6), \, \Theta ^{\prime \prime} = yy2$. This system of Eqs. (10) and (12) are reduced to first-order equations incorporated with boundary conditions.

$$A_{1} = \left( {1 - \psi } \right)^{2.5} \left( {1 - \psi + \psi \frac{{\rho_{s} }}{{\rho_{f} }}} \right){\text{, A}}_{2} = (1 - \psi + \psi \frac{{(\rho C_{p} )_{p} }}{{(\rho C_{p} )_{f} }}),{\text{ A}}_{3} = \left( {1 - \psi } \right)^{2.5} ,$$

19 $$\begin{aligned} yy1 &= - \tfrac{2}{{\xi + k_{c} }}y(4) + \tfrac{1}{{(\xi + k_{c} )^{2} }}y(3) - \tfrac{1}{{(\xi + k_{c} )^{3} }}y(2) - A_{1} \left[ \begin{aligned} & \tfrac{{k_{c} }}{{\xi + k_{c} }}(y(2)y(3) - y(1)y(4)) \\ & \quad\quad\quad - \tfrac{{k_{c} }}{{(\xi + k_{c} )^{2} }}(y(2)^{2} - y(1)y(3)) \\ & - \tfrac{{k_{c} }}{{(\xi + k_{c} )^{3} }}y(1)y(2) \end{aligned} \right] \\  + A_{3} M\left( {y(3) + \tfrac{1}{{\xi + k_{c} }}y(2)} \right), \\ \end{aligned} $$

20 $$\begin{aligned} yy2 &= - \frac {\Pr \left[ \begin{gathered} 3Ra(1 + (\Theta_{W} - 1)y(5))^{2} (\Theta_{W} - 1)y(6)^{2} { + }\lambda^{*} y(5){ + } \hfill \\ A_{3} \left( {\frac{{k_{c} }}{{\xi + k_{c} }}\left\{ {y(1)y(6) + y(2)y(5) + \frac{{S_{t} }}{{1 - S_{t} }}y(2)} \right\}} \right) \hfill \\ \end{gathered} \right]}{(\frac{{k_{nf} }}{{k_{f} }} + Ra(1 + (\Theta_{W} - 1)y(5))^{3} )}  - \frac{1}{{\xi + k_{c} }}y(6), \\ \end{aligned}$$

with

21 $${\text{y}}_{0} {(1)};{\text{ y}}_{0} (2) - 1 - \kappa \left( {y_{0} (3) + \frac{1}{{k_{c} }}y_{0} (2)} \right){\text{; y}}(6) - 1{ + }S_{t} ;\,y_{\inf } (2);\,y_{\inf } (3);\,y_{\inf } {(5)}{\text{.}}$$

Suitable selected initial guesses are supposed to satisfy the boundary conditions and the tolerance for the problem under consideration is taken as $10^{ - 6}$. The chosen primary estimate must correlate with the boundary condition asymptotically and the solution as well. Depending on the values of the engaged parameters, we have utilized appropriate finite estimates of $\xi \to \infty$, for computing the numerical solution.

## Results and discussion

**Figure 2:**
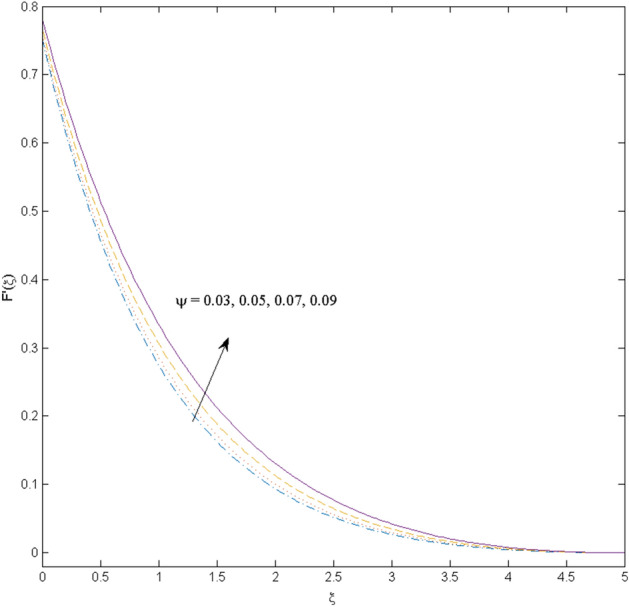
Velocity profile $F^{{\prime }} (\xi )$ influenced by $\psi$.

**Figure 3:**
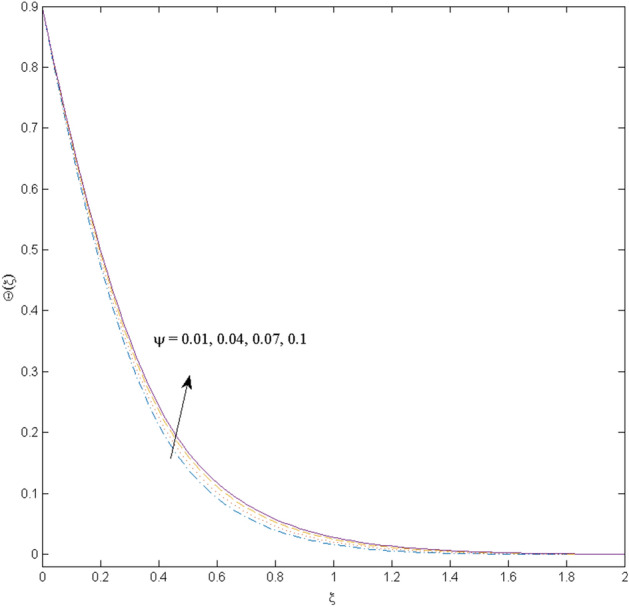
Temperature profile $\Theta (\xi )$ influenced by $\psi$.

**Figure 4:**
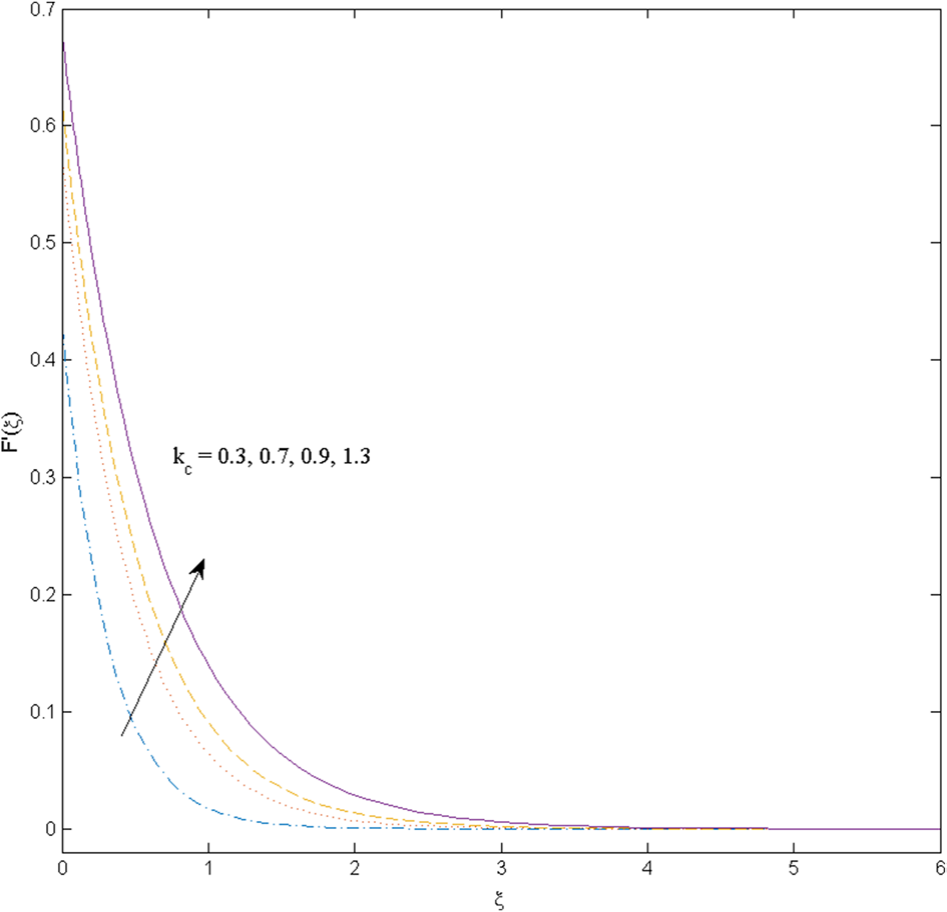
Velocity profile $F^{{\prime }} (\xi )$ influenced by $k_{c}$.

**Figure 5:**
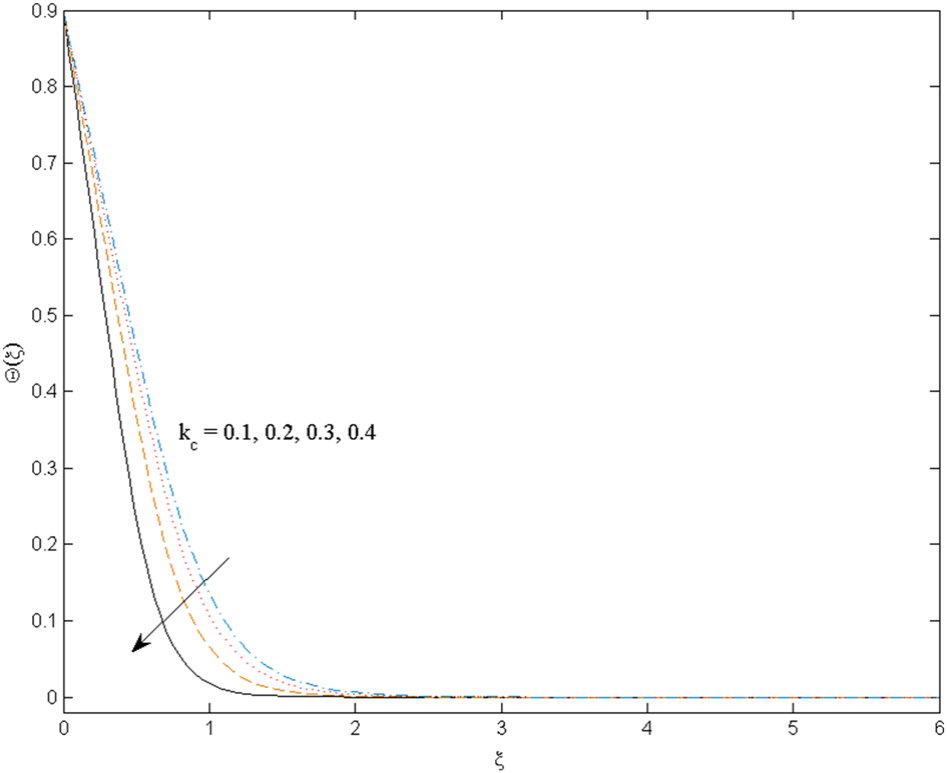
Temperature profile $\Theta (\xi )$ influenced by $k_{c}$.

**Figure 6:**
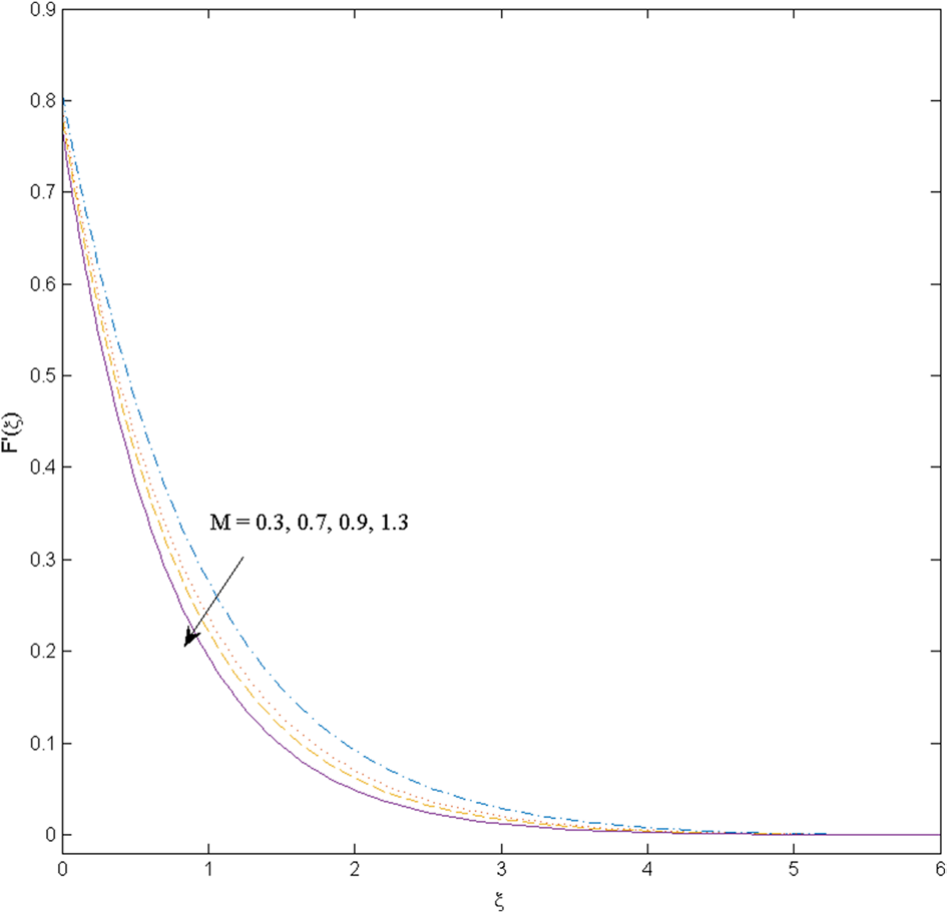
Velocity profile $F^{{\prime }} (\xi )$ influenced by $M$.

**Figure 7:**
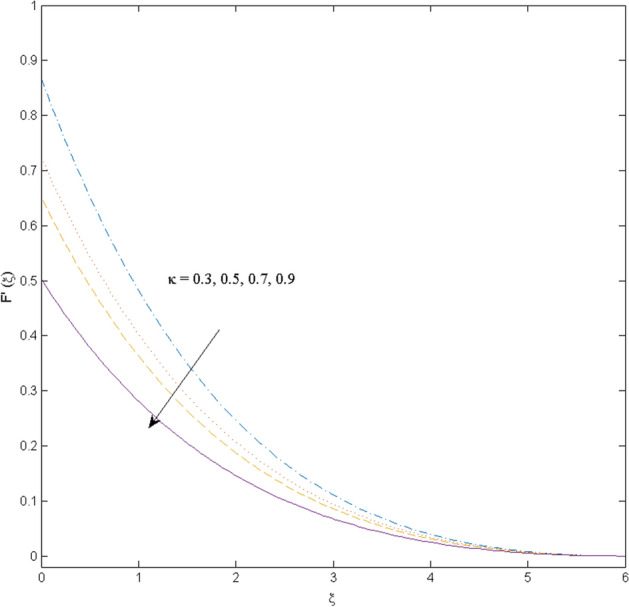
Velocity profile $F^{{\prime }} (\xi )$ influenced by $\kappa$.

**Figure 8:**
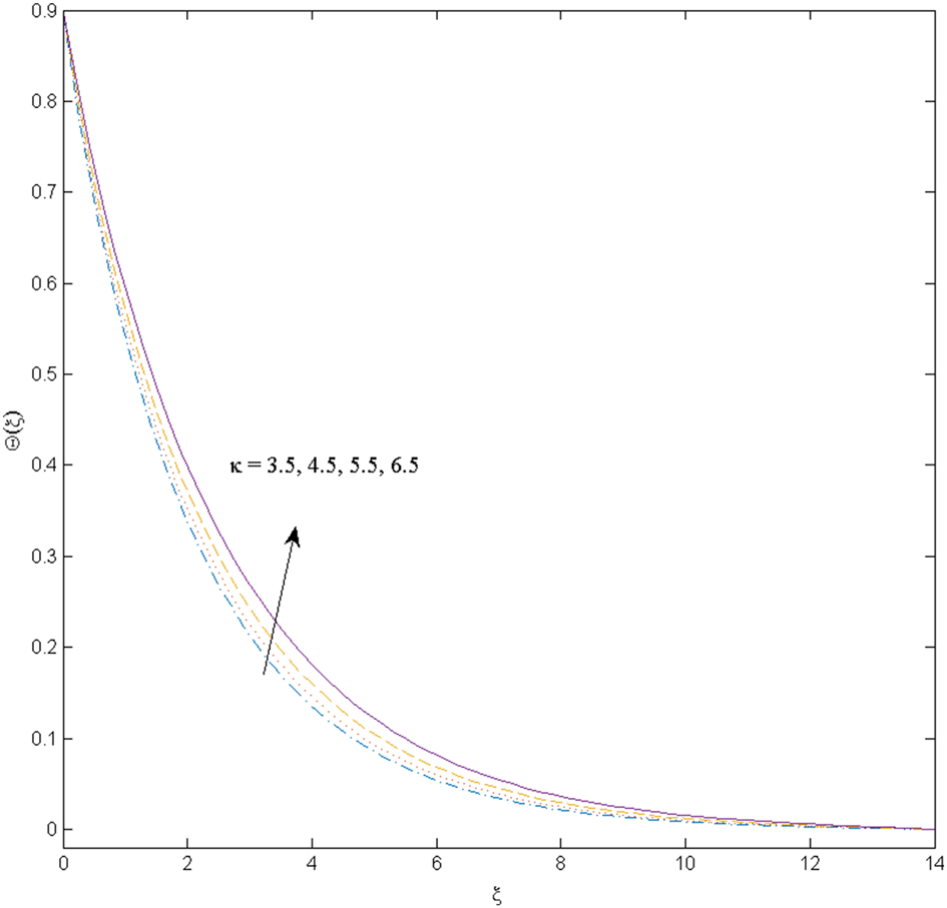
Temperature profile $\Theta (\xi )$ influenced by $\kappa$.

**Figure 9:**
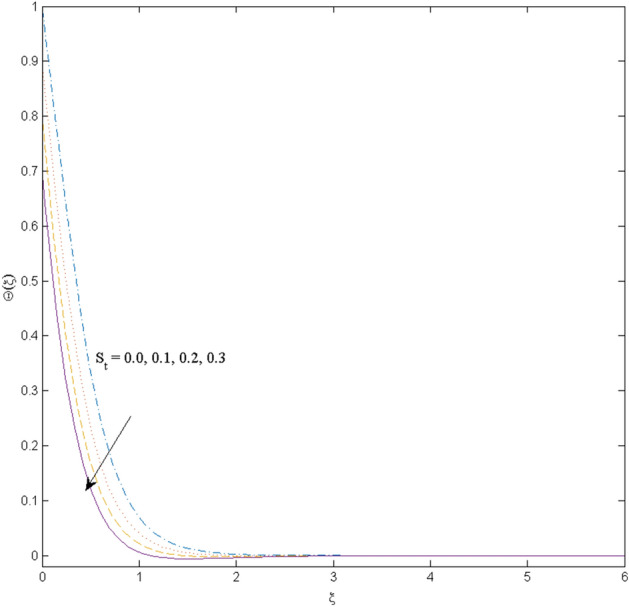
Temperature profile $\Theta (\xi )$ influenced by $S_{t}$.

**Figure 10:**
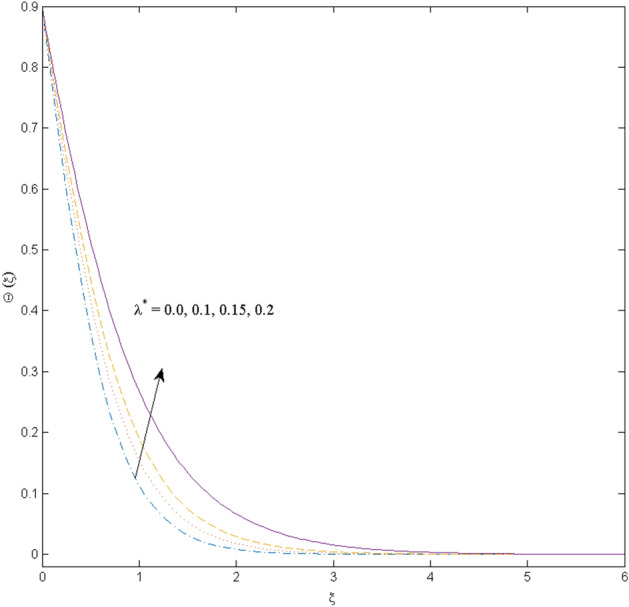
Temperature profile $\Theta (\xi )$ influenced by $\lambda^{*}$.

**Figure 11:**
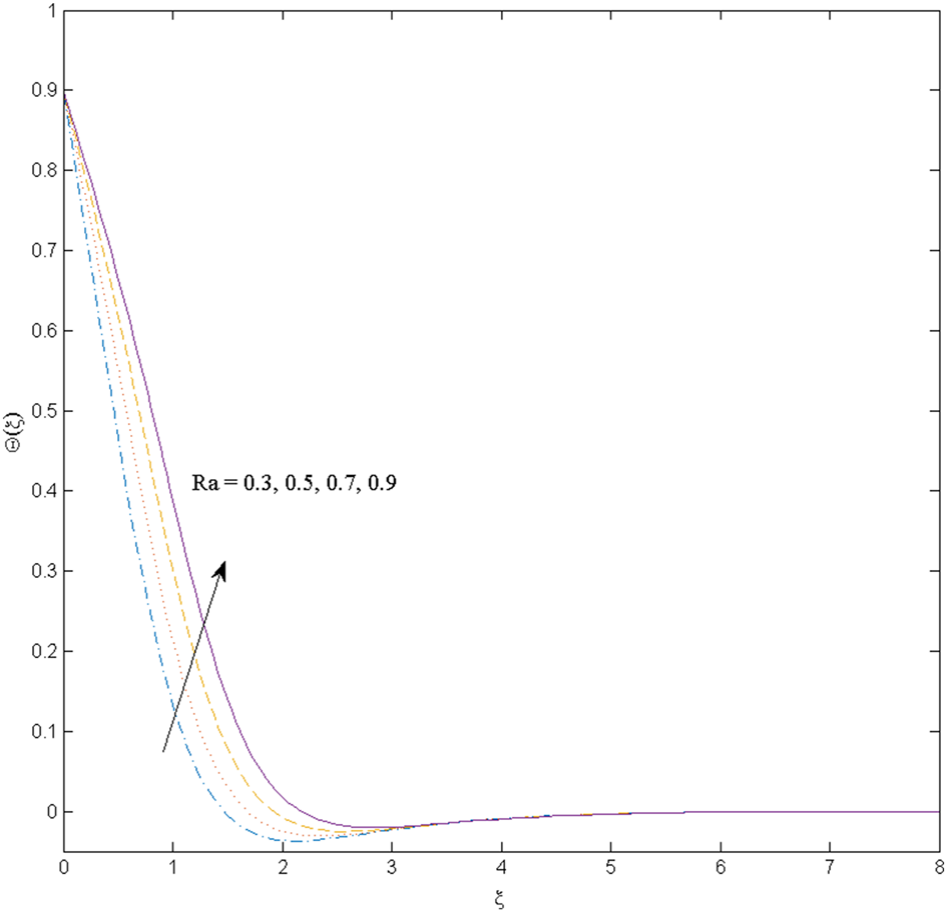
Temperature profile $\Theta (\xi )$ influenced by $Ra$.

**Figure 12:**
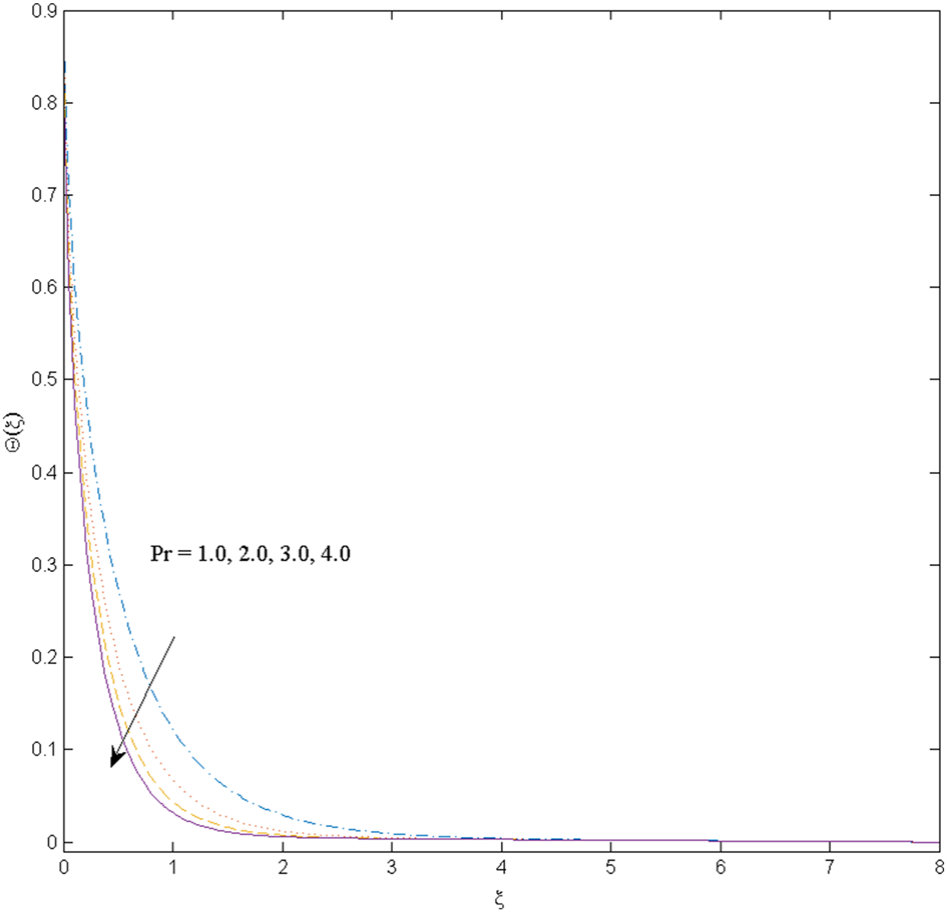
Temperature profile $\Theta (\xi )$ influenced by $\Pr$.

This section (Figs. 2, 3, 4, 5, 6, 7, 8, 9, 10, 11, 12) aims to deliberate the impacts of numerous arising parameters including radiation parameter, magnetic number, slip parameter, heat generation/absorption parameter, stratification parameter, solid volume fraction and Prandtl number versus velocity, and the temperature profiles. The values of the parameters are fixed as $M = \psi = S_{t} = 0.1,$ $\lambda^{*} = \Theta_{W} = Ra = 0.5,$ $k_{c} = \Pr = 10$ and κ = 0.2, otherwise stated. Figures 2 and 3 are drawn to observe the impression of nanoparticle volume fraction $\psi$ on velocity and temperature distributions. An upsurge in the values of $\psi$ results in an increase in both velocity and temperature fields. Large estimates of $\psi$ lead to higher thermal conductivity which increases the fluid temperature. The association of the curvature parameter $k_{c}$ with the fluid velocity and temperature is given in Figs. 4 and 5 respectively. It is comprehended that the fluid velocity is an escalating function of the $k_{c}$ however an opposing trend is noted for temperature profile. Physically, a rise in the value of $k_{c}$ results in lowering the radius of the curved sheet means fluid will experience a less contact surface area and ultimately minimum resistance will be witnessed to the fluid. Thus, the velocity profile shows mounting values. Nevertheless, a surge in the radius of curvature declines the temperature distribution. Physically, the transfer of the temperature to the fluid from the surface is slower in contrast to the curved stretching sheet. Thus, a declined temperature is observed here. Figure 6 is displayed to show the correlation amid the magnetic parameter *M* with the fluid velocity. A decline in the fluid velocity is seen for rising estimates of *M*. Large values of *M* means the strong Lorentz force thus offering resistance to the fluid motion and eventually declined fluid velocity is seen. To witness the impact of the slip parameter κ on the fluid velocity and the temperature, Figs. 7 and 8 are drawn. The fluid velocity deteriorates and an opposite behavior is witnessed in the case of fluid temperature. Strong resistance is experienced in transporting the stretched velocity to the fluid owing to the frail bonding between the wall and the fluid in the presence of slip. That is why fluid velocity deteriorates. In the case of temperature, more heat is transmuted to the fluid from the surface wall because of strong friction. Thus, fluid temperature escalates. Figure 9 depicts the variation in the fluid temperature versus the stratification parameter $S_{t}$. A downfall in the fluid temperature is witnessed for escalating estimates of $S_{t}$. As the values of the stratification parameter mount, the difference of ambient and surface temperatures is minimized. This causes a thinning boundary layer which implies a reduction in fluid temperature. Furthermore, it is pertinent to mention that $S_{t} = 0$, symbolize the prescribed surface temperature. The behavior of the heat generation/absorption parameter $\lambda^{*}$ is portrayed in Fig. 10. Large estimates of $\lambda^{*}$ causes fluid temperature to rise. Physically, this is due to the presence of the external heat source term and heat energy generation in fluid particles that boots the fluid temperature. Figure 11 is outlined to depict the impression of the nonlinear thermal radiation parameter *Ra* on the temperature profile. An upsurge in the fluid temperature is observed for varied estimates of *Ra*. As the radiation parameter is augmented, the absorbed heat from the heated plates is transferred to the fluid and ultimately an escalation in fluid temperature is encountered. The outcome of the Prandtl number Pr versus temperature profile is portrayed in Fig. 12. A downfall in the fluid temperature is noted for growing estimates of Pr. Higher estimates of Pr means the weaker thermal diffusivity. Thus, affecting the fluid temperature.

Table 2 is illustrated to validate the presented results. an exceptional concurrence with Sanni et al.^46^ and Lu et al.^51^ is achieved when m = 1, $\psi$ = 0.0. Table 3 depicts the outcome of numerous parameters on the Skin friction coefficient $- \frac{1}{2}(Re_{x} )^{0.5} C_{fx}$ and the Nusselt number $(Re_{x} )^{ - 0.5} Nu_{x}$. It is observed that the drag force coefficient grows for large estimates of the magnetic parameter and the nanoparticle volume fraction, however, it declines to owe to rising estimates of slip and curvature parameters. It also noticed that the drag force coefficient shows a decline impression when the value of the radius of curvature increases. High estimates of the radius of curvature affect the radius thus damaging the fluid resistance. As observed the Nusselt number dominates for higher estimates of solid volume fraction, radiation parameter, and it declines for rising values of slip, magnetic, thermal stratification, heat generation, and curvature parameters. It is examined that a rise in the value of the radiation parameter elevates the magnitude of the rate of heat flux which eventually increases the heat transfer rate. It is also noticed that the escalating values of the radius of curvature cause a decrease in the magnitude of local Nussselt number since the decrease in radius of curvature produces an increase in fluid temperature. The heat flux show escalating behavior for the rising values of stratification. It is due to the fall in temperature difference between the ambient and surface temperatures of the curve.

**Table 2:** Results comparison, for Skin friction coefficient $\frac{1}{2}(Re_{x} )^{0.5} C_{fx}$ when m = 1 and $\psi = 0.0$.

$k_{c}$	Sanni et al.^46^	Lu et al.^51^	Current result
5	1.1576	1.15760	1.15761
10	1.0734	1.07341	1.07340
20	1.0355	1.03540	1.03544
50	1.0140	1.01400	1.01400
100	1.0070	1.0069	1.00703
1000	1.0008	1.00079	1.00080

**Table 3:** Numerical outcomes of the skin friction coefficient $- \frac{1}{2}(Re_{x} )^{0.5} C_{fx}$ and Nusselt number $(Re_{x} )^{ - 0.5} Nu_{x}$ for different value of $\kappa$, $S_{t}$, $\lambda^{*}$_,_ $\Pr$, $Ra$, $M$_,_ $\psi$ and $k_{c}$.

$\kappa$	$S_{t}$	$\lambda^{*}$	$\Pr$	$Ra$	$M$	$\psi$	$k_{c}$	$- \frac{1}{2}(Re_{x} )^{0.5} C_{fx}$	$(Re_{x} )^{ - 0.5} Nu_{x}$
0.1	0.1	0.5	10	0.5	0.1	0.1	10	1.3291553	4.7740485
0.2								1.1728782	4.2556719
0.3								1.0526318	3.7670128
0.1	0.2							1.3291553	4.6840764
	0.3							1.3291553	4.6405272
	0.4							1.3291553	4.6350093
	0.1	0.1						1.3291553	6.6834651
		0.2						1.3291553	6.2755559
		0.3						1.3291553	5.8318231
		0.5	0.1					1.3291553	0.38983056
			0.2					1.3291553	0.28339043
			0.3					1.3291553	0.08708853
			10	0.2				1.3291553	4.1672184
				0.3				1.3291553	4.3855480
				0.4				1.3291553	4.5881019
				0.5	0.2			1.3668642	4.7011298
					0.3			1.4032186	4.6266452
					0.4			1.4383243	4.5498593
					0.1	0.2		1.7288174	5.0628829
						0.3		2.2802923	5.6515857
						0.4		3.0923874	6.3707609
						0.1	1	2.8818797	9.4400293
							2	2.0129193	5.0363786
							3	1.7076896	3.7962643

Summing up the above discussion it is comprehended from Table 2 that the surface drag coefficient and the rate of heat transfer exhibit a diminishing trend for the slip and radius of curvature parameters. Nevertheless, both show an escalating tendency for nanoparticle volume fraction. In the case of the magnetic parameter, an opposing trend is noticed for the surface drag coefficient and the heat transfer rate.

## Final remarks

In the present exploration, we have studied the flow of nanofluid containing Nickel-Zinc Ferrite and Ethylene glycol over a curved surface with heat transfer analysis. The heat equation includes nonlinear thermal radiation and heat generation/absorption effects. The proposed mathematical model is reinforced by the slip and the thermal stratification boundary conditions. Pertinent transformations are engaged to attain the system of ordinary differential equations from the governing system in curvilinear coordinates. A numerical solution is uncovered by applying MATLAB build-in function bvp4c. Depiction via graphical illustrations and numerically erected tabulated values with essential discussions explains the impacts of several arising parameters on concerned profiles. However, the computational methods used in this work are not suitable to calculate solutions for higher Prandtl numbers. The presented results are intended to show trends of each variable, but for technical applications more realistic Prandtl numbers need to be explored. The present attempt possesses the subsequent salient features:

- The velocity distribution declines due to increment in the slip parameter.
- The temperature grows for large estimates of the heat generation/absorption parameter.
- The fluid velocity is enhanced for mounting values of the curvature parameter however an opposing trend is noted for the temperature profile
- An increase in temperature is witnessed for large estimates of thermal radiation parameter but a differing trend is seen for growing values of the stratification parameter.
- The surface drag force coefficient enhances for a strong magnetic field and a decline is observed for the slip and radius of curvature parameters.
- The rate of heat transfer escalates for radiation parameter and plummets for growing estimates of stratification, slip, and radius of curvature parameters.

## List of symbols

$$C_{f}$$: Skin friction
$F$: Dimensionless stream function
*k*: Thermal conductivity
$k_{c}$: Radius of curvature parameter
$\bar{k}$: Mean absorption coefficient
*L*: Slip length
*M*: Magnetic field parameter
$Nu_{x}$: Local Nusselt number
$$n_{1} ,n_{2}$$: Constant
*P*: Dimensionless Fluid pressure
Pr: Prandtl number
$Q^{*}$: Volumetric rate of heat generation
$q_{r}$: Nonlinear radiative heat flux
$q_{W}$: Wall’s heat flux
$$Ra$$: Radiation parameter
*r*: Curvilinear coordinate
$\bar{R}$: Radius of curve
*S*: Stretching constant
$$S_{t}$$: Thermal stratification
$T_{W}$: Temperature at the sheet
$T_{0}$: Upper wall temperature
$$T,T_{\infty }$$: Temperature
$U_{W}$: Stretching velocity along x-direction
*U*: Velocity component in *r*-plane
*V*: Velocity component in *x*-plane
*x*: Curvilinear coordinate

## Greek symbols

$\alpha$: Modified thermal diffusivity
$\beta_{0}$: Strength of magnetic field
$\kappa$: Dimensionless slip parameter
$\lambda^{*}$: Heat generation parameter
$\mu$: Dynamic viscosity (kg m^−1^ s^−1^)
$$\upsilon$$: Kinematic viscosity
$\psi$: Nanoparticle volume fraction
$\rho$: Mass density (kg m^−3^)
$\rho C_{p}$: Heat capacity (kg m^−1^ s^−2^)
$\bar{\sigma }$: Stefan Boltzmann constant
$$\sigma *$$: Electrical conductivity (S m^−1^)
$\tau_{rx}$: Shear stress in $rx$-plane
$$\Theta$$: Dimensionless temperature
$\Theta_{W}$: Temperature ratio parameter
$$\xi$$: Similarity variable

## Subscripts

*f*: The base fluid
*nf*: The nanofluid
*p*: The nanoparticles
*rr*: Partial derivate w.r.t *r*
*r*: Partial derivate w.r.t *r*
*s*: Nano-solid-particles
*W*: For wall surface
*x*: Partial derivate w.r.t *x*
$$\infty$$: For ambient
