# Author Correction: Numerical treatment of radiative Nickel–Zinc ferrite-Ethylene glycol nanofluid flow past a curved surface with thermal stratification and slip conditions

**DOI:** 10.1038/s41598-025-98336-x

**Published:** 2025-05-08

**Authors:** Muhammad Ramzan, Nosheen Gul, Jae Dong Chung, Seifedine Kadry, Yu-Ming Chu

**Affiliations:** 1 Department of Computer Science, Bahria University, Islamabad Campus, Islamabad, 44000 Pakistan; 2 Department of Mechanical Engineering, Sejong University, Seoul, 143-747 Korea; 3 Department of Mathematics and Computer Science, Faculty of Science, Beirut Arab University, Beirut, 115020 Lebanon; 4 Department of Mathematics, Huzhou University, Huzhou, 313000 People’s Republic of China; 5 Hunan Provincial Key Laboratory of Mathematical Modeling and Analysis in Engineering, Changsha University of Science and Technology, Changsha, 410114 People’s Republic of China

Correction to: *Scientific Reports* 10.1038/s41598-020-73720-x, published online 08 October 2020

The original version of this Article contained an error, where a sentence was omitted in the paragraph under the section “Final remarks”:

“In the present exploration, we have studied the flow of nanofluid containing Nickel-Zinc Ferrite and Ethylene glycol over a curved surface with heat transfer analysis. The heat equation includes nonlinear thermal radiation and heat generation/absorption effects. The proposed mathematical model is reinforced by the slip and the thermal stratification boundary conditions. Pertinent transformations are engaged to attain the system of ordinary differential equations from the governing system in curvilinear coordinates. A numerical solution is uncovered by applying MATLAB build-in function bvp4c. Depiction via graphical illustrations and numerically erected tabulated values with essential discussions explains the impacts of several arising parameters on concerned profiles. The present attempt possesses the subsequent salient features:”

Now reads:

“In the present exploration, we have studied the flow of nanofluid containing Nickel-Zinc Ferrite and Ethylene glycol over a curved surface with heat transfer analysis. The heat equation includes nonlinear thermal radiation and heat generation/absorption effects. The proposed mathematical model is reinforced by the slip and the thermal stratification boundary conditions. Pertinent transformations are engaged to attain the system of ordinary differential equations from the governing system in curvilinear coordinates. A numerical solution is uncovered by applying MATLAB build-in function bvp4c. Depiction via graphical illustrations and numerically erected tabulated values with essential discussions explains the impacts of several arising parameters on concerned profiles. However, the computational methods used in this work are not suitable to calculate solutions for higher Prandtl numbers. The presented results are intended to show trends of each variable, but for technical applications more realistic Prandtl numbers need to be explored. The present attempt possesses the subsequent salient features:”

The original article has been corrected.

